# Introducing Materials Science: Experimenting with Magnetic Nanomaterials in the Undergraduate Chemistry Laboratory

**DOI:** 10.1021/acs.jchemed.3c00121

**Published:** 2023-05-08

**Authors:** Annie Regan, John O’Donoghue, Carl Poree, Peter W. Dunne

**Affiliations:** † School of Chemistry, Trinity College Dublin, College Green, Dublin D02 PN40, Ireland; ‡ CDT ACM, AMBER, Trinity College Dublin, College Green, Dublin D02 PN40, Ireland

**Keywords:** Upper-Division Undergraduate, Inorganic Chemistry, Laboratory Instruction, Hands-On Learning, Magnetic Properties, Materials Science, Synthesis, X-ray Crystallography

## Abstract

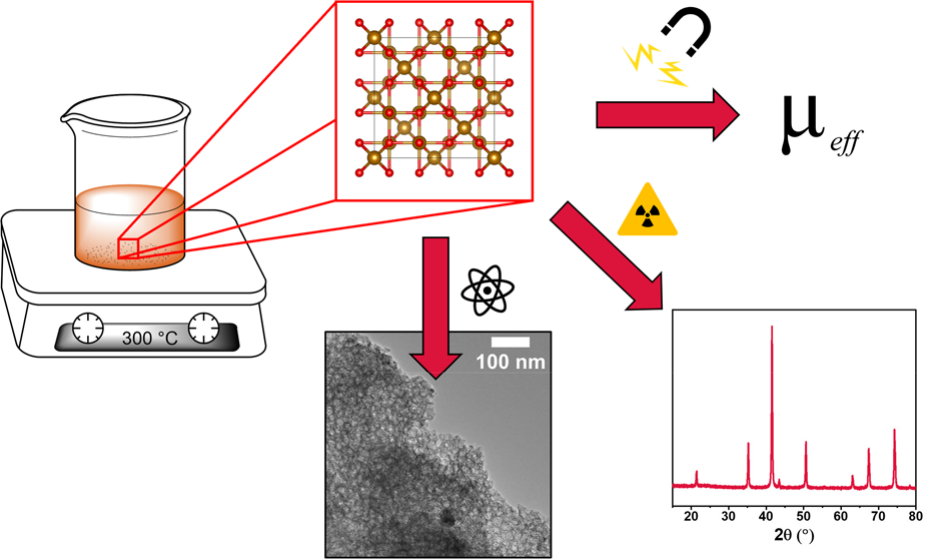

Materials science research has expanded significantly in recent years; a multidisciplinary field, home to an ever-growing number of chemists. However, our general chemistry degree courses have not changed to reflect the rise in interest in this topic. In this paper, we propose a laboratory experiment for the undergraduate chemistry practical course, which may serve as a hands-on introduction to this field. The experiment involves the synthesis and characterization of magnetic materials via commonly employed techniques in materials science. Students begin by producing three metal ferrite spinels using a sol–gel combustion synthesis. They must then characterize the differing magnetic properties across their three samples using a magnetic susceptibility balance. In the second part of the experiment, students must create a ferrofluid via coprecipitation, from which they may observe the phenomenon of “spiking” in response to an external magnet. Additional data such as X-ray diffraction (XRD) patterns and transmission electron microscopy (TEM) images corresponding to these materials are also provided, and students are tasked with the interpretation of these data in their writeup report. Upon completion, students should gain a new-found understanding of materials science and its fundamental overlap with chemistry.

## Introduction

From the beginnings of the Stone age to the modern-day era of graphene fanfare, materials science has been a crucial area of research and engineering since long before the term had been coined. Fundamentally, it comprises the study of material properties and how these can be manipulated by changes in structure and surface chemistry. As such, a broad spectrum of expertise is required to fully understand and characterize these materials; from the physics underpinning their atomic structures and properties, to the chemistry behind their formation and behavior, and finally to engineering said materials for practical use on a global scale. While materials science has been recognized as its own field since the 1950s,^1^ the term is not as commonplace among students entering higher education as the core sciences of chemistry and physics, nor is it as widely offered as a specialized discipline of undergraduate study. Instead, the responsibility must fall on higher level institutions and educators to introduce this field to students studying related areas, e.g., over the course of their physics, chemistry, or engineering degrees. As of yet, little mention is given to this topic in pure chemistry courses, despite its fundamental overlap with inorganic chemistry, in particular.^2^ Instead, topics such as transition metal complexes and coordination chemistry are seen to dominate inorganic teaching across the board, while few institutions purport to include materials science or its related topics in their chemistry discipline at any undergraduate level.^3,4^

In Trinity College Dublin (TCD) in 2020, the inorganic chemistry practical course taken by undergraduate students in their penultimate year of chemical sciences was adapted to include a new experiment, designed to introduce topics and techniques more typically encountered in the field of materials science. While there are some valuable examples in the literature of experiments designed for this purpose, they are typically introduced as an “aside” to other synthetic techniques, with a general emphasis on various materials’ applications rather than the underpinning theory.^5−9^ The experiment introduced here, entitled “Magnetic Nanomaterials”, is a practical centered around the synthesis and characterization of a selection of magnetic powders and a ferrofluid via sol–gel combustion synthesis and coprecipitation. It is designed to bridge the conceptual gap between practical aspects and theory by applying familiar inorganic chemistry concepts such as coordination chemistry and crystal field theory to rationalize materials’ properties. As well as tying in these fundamental inorganic chemistry concepts, this experiment purposely introduces other characterization techniques such as powder XRD and TEM, which are very rarely encountered in an inorganic practical course in comparison to spectroscopic methods.^9,10^

**Figure 1:**
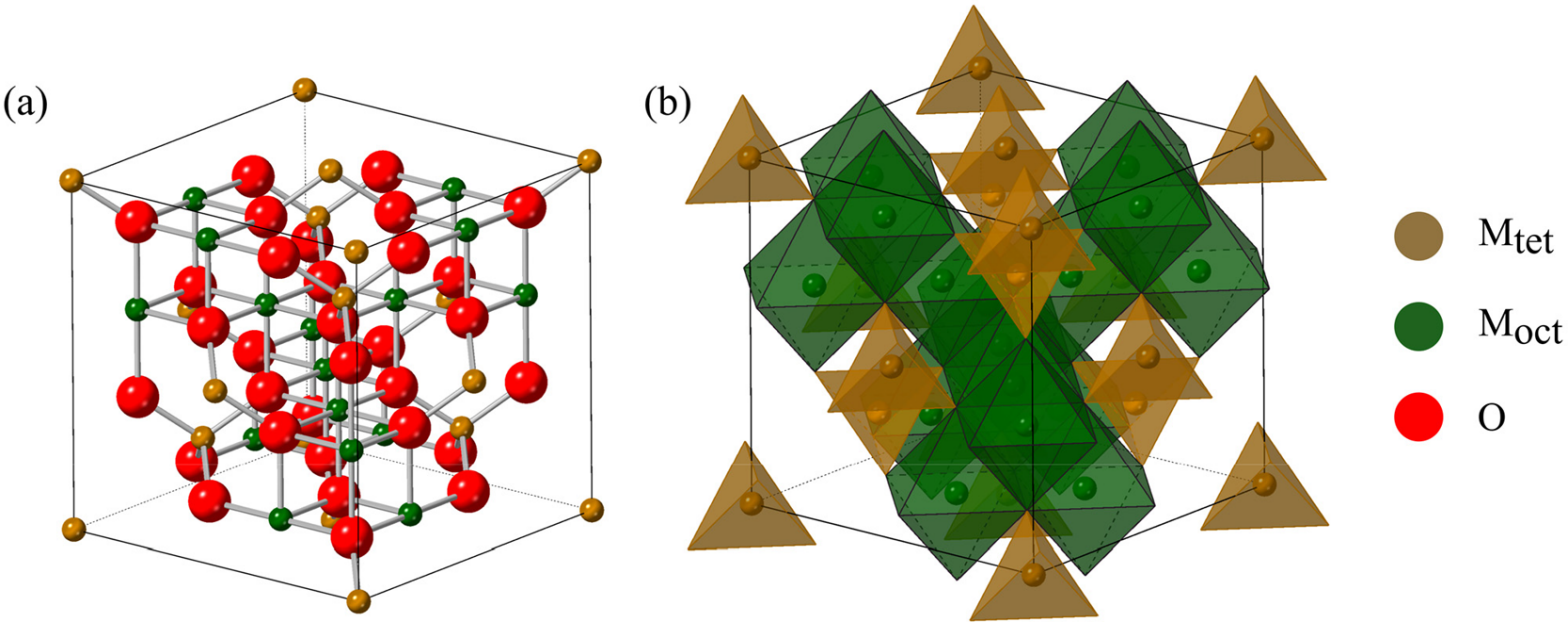
(a) Unit cell of magnetite, Fe_3_O_4_, and (b) polyhedral model highlighting the octahedral and tetrahedral sites.

One of the major properties of interest and practicality in materials science is that of magnetism; with applications ranging from data storage to biomedical imaging.^11,12^ Possibly the most widely used materials for such applications are iron oxide and its related spinel ferrites, with the general formula MFe_2_O_4_, where M = Fe^2+^, Co^2+^, Ni^2+^, Zn^2+^. Spinel ferrites are of particular interest due to their compositional flexibility, as a wide range of different magnetic properties can be accessed by variation of the metal ions and structure. The spinel structure may adopt one of two configurations; normal or inverse, depending on the most energetically favorable configuration of electron spins in the lattice. In the normal spinel structure, the M^2+^ ions occupy 1/8 of the tetrahedral sites and the Fe^3+^ ions occupy 1/2 of the octahedral sites in the cubic close packed oxide array, while for the inverse case the M^2+^ ions swap places with half of the Fe^3+^ ions, as shown in Figure 1 for the structure of magnetite.

**Figure 2:**
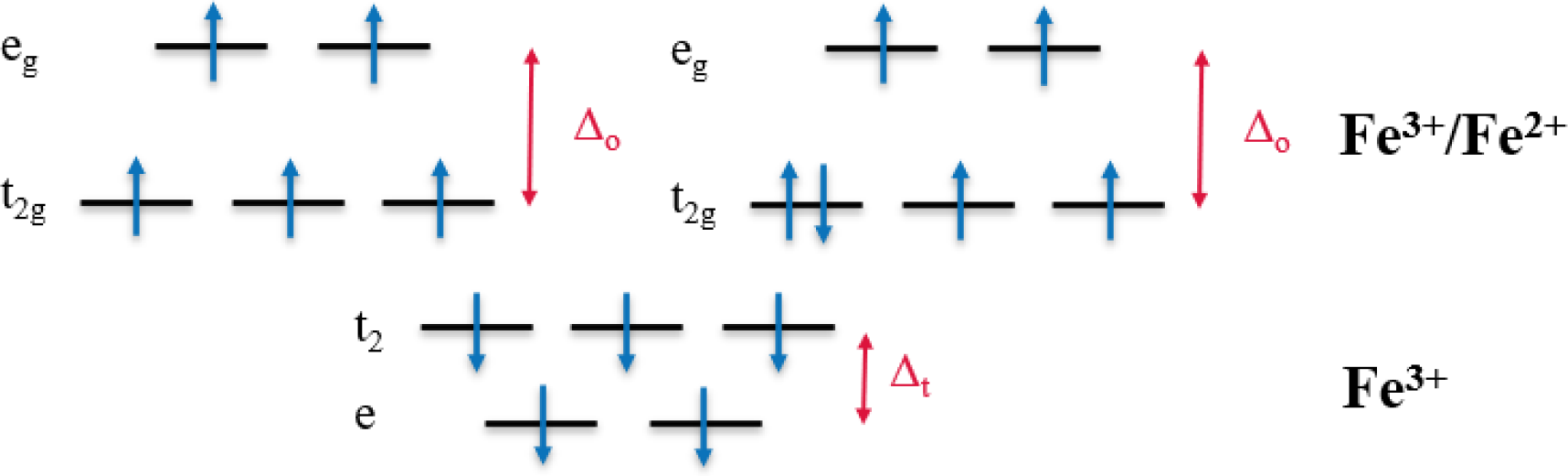
Crystal field splitting diagram showing the ordering of spins in magnetite, Fe^II^Fe^III^_2_O_4_, demonstrating the number of unpaired electrons and the filling of Fe^II^ ions in the octahedral sites according to the inverse spinel structure.

This in turn also determines the magnetic characteristics of the material, whereby normal spinels exhibit antiferromagnetism, and the inverse exhibit ferromagnetism, as described by the Goodenough-Kanamori rules.^13,14^ One can predict which type of structure should be adopted by a given spinel (and thus its magnetic properties) based on a calculation of the crystal field stabilization energy (CFSE) resulting from each possible ordering of spins across the octahedral and tetrahedral sites, e.g., for magnetite (Fe^II^Fe^III^_2_O_4_):
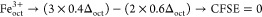

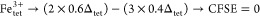

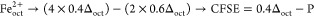

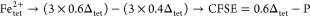
where Δ_oct_ is the octahedral field stabilization energy, Δ_tet_ the tetrahedral field stabilization energy, and *P* the spin pairing energy. Note that Δ_oct_ is ×2.25 Δ_tet_, and so while there is no preference for where the Fe^3+^ ions reside, the CFSE is maximized when Fe^2+^ ions solely occupy the octahedral sites in the lattice. This yields an inverse spinel structure for magnetite, visualized in Figure 2, which shows the arrangement of d-electrons across both sites. Note that in the octahedral field, electrons sit across *e*_*g*_ and *t*_2*g*_ sites according to which lobes of the d-orbitals they originate from, whereby *d*_*z*^2^_ and *d*_*x*^2^–*y*^2^_ orbitals are more destabilized than the *d*_*xy*_, *d*_*xz*_, and *d*_*yz*_ orbitals as the axial oxygen atoms interact more strongly than those situated equatorially. In the tetrahedral field, the reverse is true as the orbitals are directed along the axes, but the ligands are not, resulting in poor overlap between the iron and oxygen orbitals. This can similarly be demonstrated for nickel ferrite (NiFe_2_O_4_) and cobalt ferrite (CoFe_2_O_4_) which are also inverse spinels.

For zinc ferrite (ZnFe_2_O_4_), the normal spinel structure is favored, as there is no energetic preference for which sites the Fe^3+^ or Zn^2+^ ions populate. Instead, the smaller cation, Zn^2+^, is seen to occupy the smaller tetrahedral sites, with only Fe^3+^ occupying octahedral sites.^15^

## Background

To better understand these differences in magnetic properties across related materials, students are asked to prepare a range of spinel type oxides by two commonplace synthetic techniques in materials science; sol–gel and coprecipitation, and then must assess their magnetic and structural properties. A slight spin on the traditional technique, sol–gel combustion is a (relatively) low temperature, self-propagating method of materials synthesis that involves a spontaneous exothermic redox reaction between a metal nitrate salt and an organic fuel to produce a metal oxide. The reaction combines sol–gel chemistry, which typically yields poorly crystalline products, with combustion; using the heat produced from the reaction of the oxidant (nitrate) and reductant (organic fuel) coming into contact in air to drive crystallization. The resultant powders are typically porous due to the liberation of gaseous products upon reaction. Suresh et al. first demonstrated the use of this method in the facile production of spinel ferrite materials.^16^ They reported the synthesis of CoFe_2_O_4_ and Ni_0.5_Zn_0.5_Fe_2_O_4_ by rapidly heating a solution of 1) cobalt(II) nitrate, or 2) nickel(II) nitrate and zinc(II) nitrate, with iron(III) nitrate and an organic fuel, oxalyl dihydrazide (ODH, C_2_H_6_N_4_O_2_), to 350 °C. This was replicated by the author and the production of these materials, as well as NiFe_2_O_4_, and ZnFe_2_O_4_, was also found to be possible at a considerably lower combustion temperature of 190 °C, and with the use of a cheaper, less hazardous fuel; citric acid (C_6_H_8_O_7_). When choosing a fuel for this type of reaction, calculation of stoichiometry is of critical importance to ensure complete combustion. In propellant chemistry, this is achieved when the oxidizer:fuel ratio, known as the equivalence ratio (*φ_C_*), is unity,^17^ which in turn will yield the maximum heat required to drive the exothermic reaction to completion. If *φ_C_* > 1, the reaction is said to be fuel lean, and fuel rich if *φ_C_* < 1, whereby an excess of either reactants or fuel is observed to decrease the exothermicity of the system.^18^ This ratio is calculated by balancing the elemental oxidizing and reducing valencies of the compounds utilized in combustion, as follows:
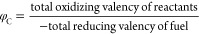
1Where the valency of each atom is taken as N → 0, O → −2*, H → +1, C → + 4, M^*n*+^ → *n*^+^ (+2, + 3, + 4, etc.).

**Figure 3:**
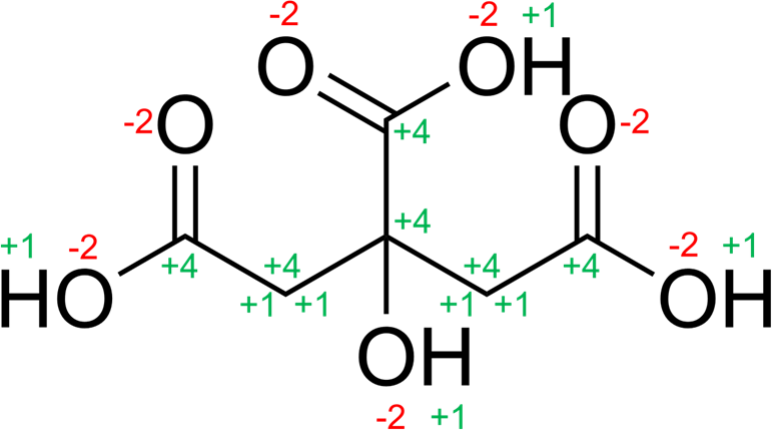
Structure of citric acid with the valency of each atom labeled to calculate the total reducing valency of the fuel. The valency of all carbons (+4 × 6), all hydrogens (+1 × 8), and all oxygens (−2 × 7) in the compound are summed to give a total valency of +18.

*Note: In this method O^2–^ is taken to be the only oxidizing element. C, H, and M^*n*+^ cations are assumed to be reducing elements, and N is assumed to be neutral.^19,20^ For the fuel citric acid (CA) this yields a total reducing valency of +18, as demonstrated in Figure 3, which is the sum of each atomic valency in the compound. With this and the calculated oxidizing valencies of metal nitrate precursors (−15 for Fe(NO_3_)_3_ and −10 for M(NO_3_)_2_, respectively), the stoichiometric ratio of reactants is determined, as shown, to maintain unity of the equivalence ratio, yielding the following balanced reaction equation:
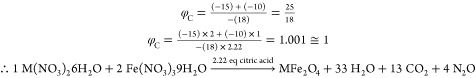
2

In the latter half of the experiment, students are introduced to the coprecipitation method; one of the most common methods of producing magnetite, Fe_3_O_4_. Coprecipitation takes advantage of the variable solubilities of species in solution, whereby water-soluble precursors are mixed in aqueous media and may be driven to form a water-insoluble product. As the concentration of product supersaturates the reaction media, precipitation takes place. Mild sol–gel coprecipitation reactions can produce sufficiently small magnetite nanoparticles of a ferromagnetic nature, which can then be readily modified with hydrophobic or hydrophilic surfactants to yield magnetic fluids in different solvents, known as ferrofluids. This part of the experiment is an adaptation of a procedure described by Berger et al.^21^ and has been included as a comparative method of synthesis to sol–gel combustion, but does not comprise an essential (or original) addition to the practical described. The main objective here is for students to (1) appreciate another common synthetic technique in materials science and (2) encounter another spinel material, exhibiting its own interesting magnetic properties.

## Student Pedagogical Goals

This experiment introduces students to commonly encountered synthetic and characterization techniques in materials science. Upon completion of this laboratory practical, students should:

- Understand the correlation and overlap between chemistry and materials science
- Obtain a deeper understanding of nanomaterials and material properties such as magnetism
- Be able to carry out sol–gel and coprecipitation syntheses
- Gain exposure to the interpretation of magnetic measurements, powder XRD, and TEM data

## Experimental Section

### Chemical Hazards and Safety Precautions

Lab coats, safety goggles, and gloves should be worn for the duration of this experiment. Students should observe the relevant hazard pictograms provided for each reagent prior to their use. Note that this experiment involves a controlled combustion, appropriate care and precautions should be taken. Ensure that all sol–gel combustion reactions are performed at the back of a well-ventilated fume hood as nitrous oxides and other noxious gases will evolve from the reacting mixture (a sufficiently large clock glass or tile should be kept nearby to cover the mouth of the beaker if the reaction becomes particularly violent). These reactions should be performed in a sufficiently oversized reaction vessel (e.g., a 500 mL beaker) to ensure the flame is controlled. Students should ensure their workspace is free of any loose tissue, papers, or other flammable materials before proceeding. In preparation of the ferrofluid, concentrated ammonia should be used only in the fume hood. Note that tetramethylammonium hydroxide is a strong base that is corrosive, flammable, and highly toxic. Remove gloves and wash hands immediately in case of contact. An alternative ligand that poses less hazard is tetrabutylammonium hydroxide,^6^ which was not selected for use in this practical due to increased cost.

### Materials

Nickel(II) nitrate hexahydrate (reagent grade) and cobalt(II) nitrate hexahydrate (99%) were obtained from Fisher Chemical. Iron(III) nitrate nonahydrate (ACS grade) was obtained from Alfa Aesar. Zinc(II) nitrate hexahydrate (98%), iron(III) chloride hexahydrate (ACS grade), citric acid (99%) and ammonium hydroxide (28–30%, ACS reagent) were obtained from Sigma-Aldrich. Iron(II) chloride tetrahydrate (≥99%) was obtained from Honeywell. Tetramethylammonium hydroxide (25 wt % in H_2_O) was obtained from Tokyo Chemical Industry. All chemicals were used without further purification.

### Procedure

#### Part 1: Spinel Ferrites

Students first prepared stock solutions of each metal nitrate precursor, and the citric acid fuel, in distilled water. The required quantities of the fuel and each metal salt were provided to students in their lab manual, to ensure the correct stoichiometric ratios were employed in the reactions. To make MFe_2_O_4_ (where M = Zn, Ni, Co) they pipetted a given volume of the relevant metal precursors into a large beaker, followed by the addition of the citric acid solution, and let the mixture stir before heating. After a few minutes, the stir bar was removed, and the hot plate set to 300 °C (to compensate for unavoidable heat loss from large, open reaction vessel). Sufficient time for reaction completion (approximately 25 min) was allowed, and the beaker allowed to cool before collecting the product. Using a pestle and mortar the product was ground down to a fine powder, and the samples were magnetically characterized using a magnetic susceptibility balance.

#### Part 2: Ferrofluid

**Figure 4:**
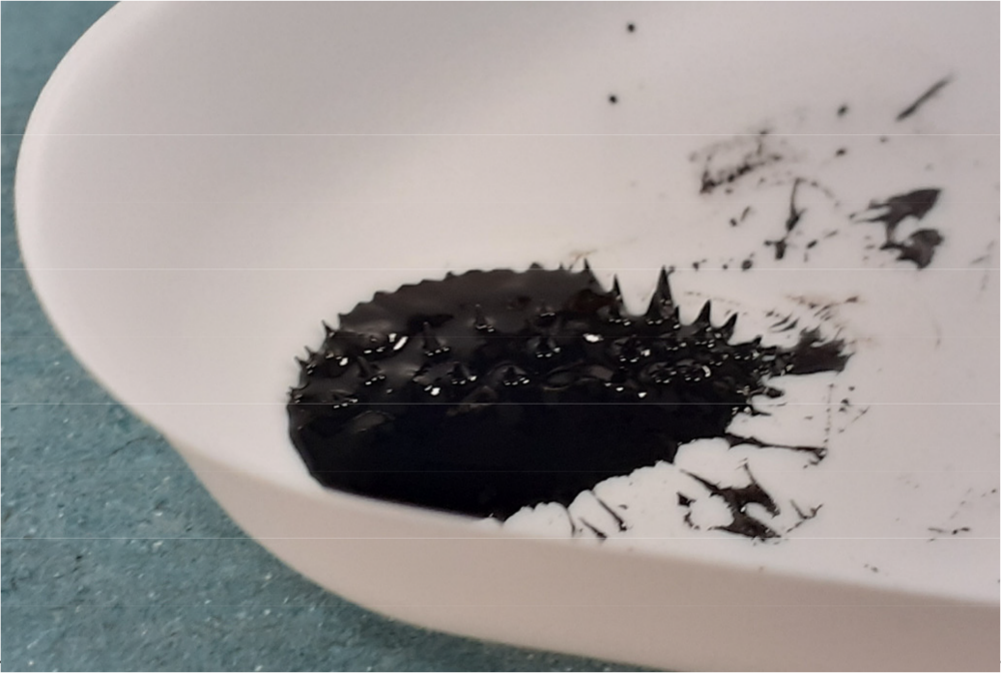
Example of a resultant ferrofluid “spiking” in response to an external magnet.

Stock solutions of iron(II) chloride tetrahydrate and iron(III) chloride hexahydrate were prepared in HCl prior to the lab class and provided to the students. To prepare the ferrofluid, students added a portion of iron(II) chloride to a given volume of iron(III) chloride. With constant stirring, approximately ∼10–13 mL of concentrated ammonia was added dropwise to the solution, resulting in a black suspension. Once the precipitate had settled, the supernatant was decanted off, and the product washed several times with distilled water. Finally, small aliquots of tetramethylammonium hydroxide (conc.) were added to the magnetite while mixing with a glass rod. Students then noted how their resultant product responded to an external magnet (e.g., see Figure 4).

### Characterization of Products

#### Magnetic Measurements using a Magnetic Susceptibility Balance

Students are asked to characterize the differences in magnetic properties of each material from part 1 by measuring the magnetic susceptibility of their samples, from which their magnetic moments can be calculated. The strongly magnetic nature of the spinel ferrites meant their readings exceeded the operating range of the instrument (a Sherwood Scientific MK 1 magnetic susceptibility balance), and so a portion of each sample was diluted with diamagnetic potassium bromide (KBr) prior to measurement (as described in the lab manual). This is accounted for in subsequent calculations using the following relationship (eq 3) to determine the mass susceptibility of the product, χ_*g*_, from the mass susceptibilities of the diluted sample and the diluent, χ_*s*_ and χ_0_, respectively. Note that *m*_0_ = mass of diluent and *m*_1_ = mass of product.
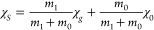
3

The remaining steps in the calculation to obtain the final magnetic moment are described in detail in the lab manual provided in the SI, performed in accordance with those described by Bain et al.^22^

#### Crystallite Size and Morphology Using XRD and TEM

As well as the magnetic measurements the students conduct themselves, they are also provided with XRD and TEM data associated with these samples to assess their structural properties. These data were collected separate to the laboratory session by the author, and the data provided to students online. It should be noted that these students have the opportunity to visit the TCD microscopy facilities for demonstrations of TEM through a separate concurrent laboratory class. Students must plot their XRD patterns with a corresponding reference pattern to verify the identity of their products, as well as fully index their diffraction patterns so as to obtain the appropriate Miller indices expected for a face centered cubic crystal system such as their spinels. They must also calculate the unit cell parameters, and the crystallite size of each sample via Scherrer analysis. This task serves as a review of material covered in an XRD lecture course and workshop completed by the students before this practical in semester 1, which demonstrated the use of analysis software such as Fityk and the use of crystallography databases to find appropriate crystallographic information files (CIFs) for this purpose.^23^ The expected results from this analysis, as well as the calculated magnetic moments for each spinel, are provided in Table 1.

**Table 1:** Properties of Spinel Ferrites Calculated from XRD and Magnetic Susceptibility Data

Sample	Unit Cell Parameter (Å)	Crystallite Size (nm)	Magnetic Moment (BM)
NiFe_2_O_4_	8.34	55	6.28
CoFe_2_O_4_	8.35	30	7.52
ZnFe_2_O_4_	8.41	20	2.78

**Figure 5:**
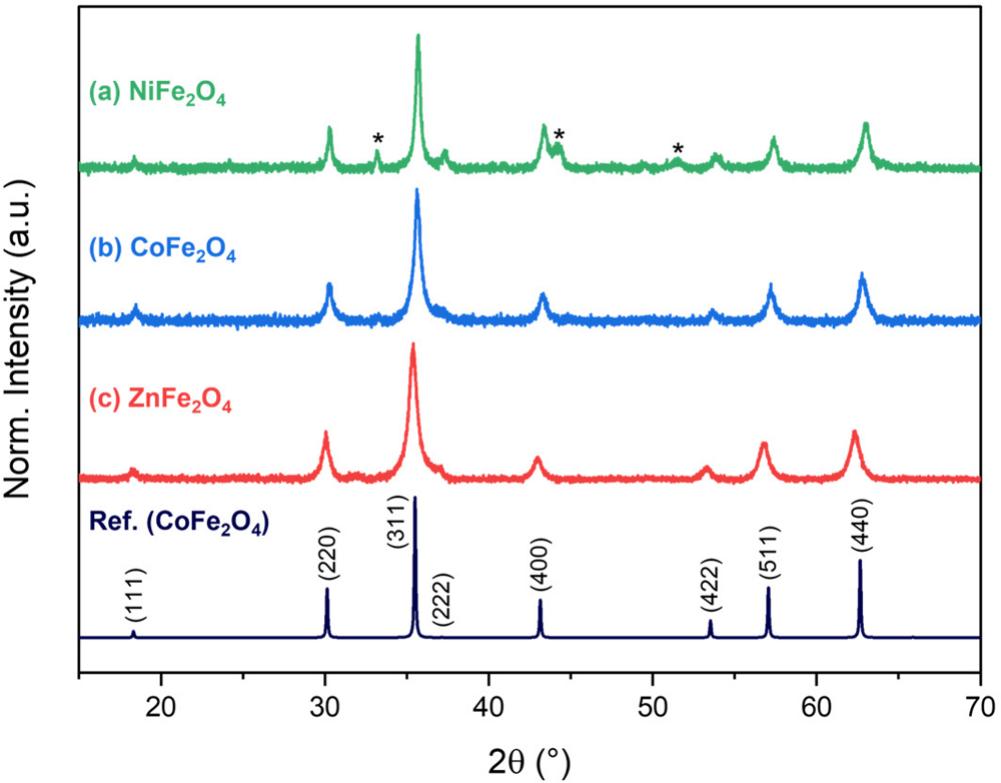
XRD patterns of the products resulting from each sol–gel combustion synthesis, with spinel reference pattern showing appropriate Miller indices for a face centered cubic crystal system (i.e., all *hkl* values are either all even or all odd). Note: Asterisk (*) indicates peaks corresponding to hematite impurity.

In Figure 5, the provided XRD patterns for each sample are shown. As indicated in pattern (a), a common impurity is known to arise from this synthesis in low concentration; hematite, α-Fe_2_O_3_, the most thermodynamically favorable phase of iron oxide. When discussing their XRD analysis, students are expected to comment on the presence of these additional peaks and use CIFs to find predicted patterns to identify this impurity, based on similar exercises carried out in XRD workshops performed previously.

**Figure 6:**
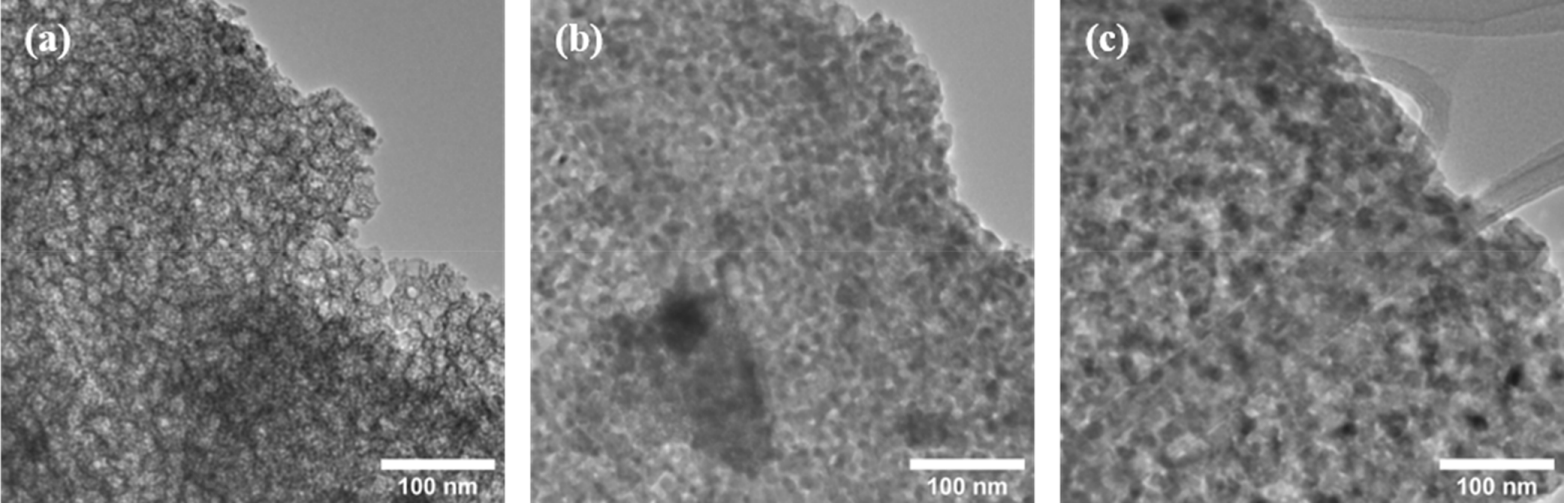
TEM micrographs showing the porosity and scale of the (a) NiFe_2_O_4_, (b) CoFe_2_O_4_, and (c) ZnFe_2_O_4_ microstructures.

As the products resulting from sol–gel combustion synthesis are porous microstructures, students cannot readily calculate a corresponding particle size from the TEM micrographs provided (Figure 6), but instead are expected to use them to qualitatively discuss the morphology and scale of their samples. Thus, in presenting their characterization data, combining XRD and TEM, students gain exposure to both numerical and qualitative structural analysis.

## Learning Outcomes

Since its addition to the course in September 2020, this experiment has been carried out in-person by a total of 68 undergraduate students in their penultimate year of studying chemical sciences at Trinity College Dublin. Students work individually and complete this experiment over the course of two separate 3 h laboratory periods, with approximately 2 weeks to write a report on the experiment, which should include a discussion of their results as well as their response to a selection of post-practical questions to test their understanding of the material covered. These reports were subsequently reviewed to determine the level of difficulty of the new practical in comparison with others on the course. It was found that an average mark of 65% was achieved, comparable to the average mark typically obtained for all other experiments in the practical course of 60–70%, which suggests this experiment has been pitched to a reasonable level of difficulty and students have adapted well to the new content being covered. The complete laboratory hand-out given to students, providing background theory and detailed pointers on what was required for their post-practical assessment, is included as Supporting Information, as well as the raw XRD and TEM data provided to them, and brief notes for instructors detailing some of the expected results of the experiment to assist with marking this assessment.

Upon completion of the 2021–22 academic year, ethics approval was granted by the TCD School of Chemistry Research Ethics Committee for an optional anonymous survey to further assess student understanding and satisfaction with the experiment. The uptake of this survey was particularly low despite multiple efforts to promote it among the students (*n* = 5, response rate = 11%). However, it did provide useful data that has contributed to the overall picture of this new experiment. When asked to provide some details of the **materials science concepts** that were used during this experiment, those who completed the survey responded with

- Magnetic Moment: Some materials have permanent magnetism due to alignment of electron dipoles.
- Crystal Field Theory: Specifically crystal field stabilization energy, X-ray diffraction.
- Spinel Crystal Structures: Predicting magnetic properties with electronic structures.
- Using the magnetic moments to determine if the spinel was normal or inverse.

When asked to describe the types of **analysis** used during the experiment, respondents said

- Mathematical comparison of octahedral preference energies of each spinel.
- Analysis of PXRD of spinels and calculation of corresponding Miller planes, lattice parameter, and crystallite size.
- Magnetic moment measurement, XRD analysis.
- Magnetic analysis, TEM, XRD.

In response to other questions in the survey, regarding difficulty and how much time was allowed for each part of the practical, survey respondents reported to have found the experiment enjoyable overall, easy to carry out or comparable in difficulty to other practicals on their course, and that they had sufficient time to complete the experiment and writeup. A copy of the survey conducted is provided in the SI.

These responses, alongside an average grade comparable to other practicals and an overall pass rate of ∼96%, would suggest that a large majority of students have successfully completed the main objectives and learning goals of the experiment; gaining insight into some of the major topics, synthetic techniques, and forms of analysis in the field of materials science. Survey responses also suggest that students have appreciated the overlap of these materials science concepts with familiar inorganic chemistry topics already covered in class, (e.g., how magnetic properties can be predicted by looking at the electronic structure of a sample using crystal field theory); a key pedagogical goal of this work.

## Conclusions

A new laboratory experiment aiming to introduce general concepts and techniques in materials science to undergraduate chemists has been added to the inorganic chemistry practical laboratories for students in their penultimate year studying chemical sciences in Trinity College Dublin (TCD). Students were found to complete both the lab and postpractical assessment to a satisfactory standard as evidenced by the grading, feedback survey, and through verbal feedback which together demonstrate that they were readily able to apply familiar chemistry concepts and theory to solve materials science problems. In TCD, students were given a total of 6 h to complete the practical and 2 weeks to complete their laboratory report. According to those surveyed, this was sufficient time to complete the practical and assessment. Students were able to complete a series of sol–gel combustion reactions to produce nickel, cobalt, and zinc ferrite spinels, as well as carrying out magnetic characterization of these samples via use of a magnetic susceptibility balance. Both via survey, and verbal feedback in the lab, students reported some difficulty in being able to produce a suitable ferrofluid that demonstrated the phenomenon of spiking but rated the difficulty of the experiment as a whole as comparable to others on their course. Overall, this practical successfully introduces some of the core topics and concepts in materials science and familiarizes undergraduate students with the field and its fundamental overlap with chemistry. In the future we would like to encourage greater uptake of the feedback survey, possibly through some form of incentive, to get a more representative spread of responses about the experiment. If possible, it would also be an invaluable addition to the experiment if students were able to be brought to the characterization facilities to see the XRD and TEM instruments first-hand.

## Supporting Information Available

Laboratory manual given to students, which provides safety considerations, background theory, post-laboratory questions, detailed instructions for performing the experiment, and instructions for computation of the magnetic measurement results (PDF)
Instructors’ notes, which briefly detail the expected results and useful notes regarding the experiment (PDF)
Student survey, which includes questions used to assess student opinion of and satisfaction with the experiment post-completion (PDF)
Raw XRD and TEM data given to students for interpretation and analysis in their lab reports (TXT, TXT, TXT, JPG, JPG, TIF)

## Supplementary Material

ed3c00121_si_006.txt

ed3c00121_si_007.jpg

ed3c00121_si_008.jpg
