# Supplementary material for: Introducing Materials Science: Experimenting with Magnetic Nanomaterials in the Undergraduate Chemistry Laboratory
Source: J Chem Educ. 2023 May 8;100(6):2387–93. doi: 10.1021/acs.jchemed.3c00121 (PMC10269328; doi:10.1021/acs.jchemed.3c00121)
Supplement: Supplementary file 1 — ed3c00121_si_001.pdf [file ed3c00121_si_001.pdf]

Supporting Information

Introducing Materials Science: Experimenting with Magnetic Nanomaterials in the Undergraduate Chemistry Laboratory

Annie Regan,[1,2] John O'Donoghue,[1] Carl Poree,[1] and Peter W. Dunne.[1] *

1. School of Chemistry, Trinity College Dublin, College Green, Dublin 2, Ireland

2. CDT ACM, AMBER, Trinity College Dublin, College Green, Dublin 2, Ireland

*Corresponding author, email: p.w.dunne@tcd.ie

Lab Manual Provided to Students

Experiment H – Magnetic Nanomaterials

Chemical hazards

Ammonia solution
Zinc(II) nitrate hexahydrate
Cobalt(II) nitrate hexahydrate
Nickel(II) nitrate hexahydrate
Iron(III) nitrate nonahydrate
Iron(II) chloride tetrahydrate
Iron(III) chloride hexahydrate
Citric acid
Tetramethylammonium hydroxide

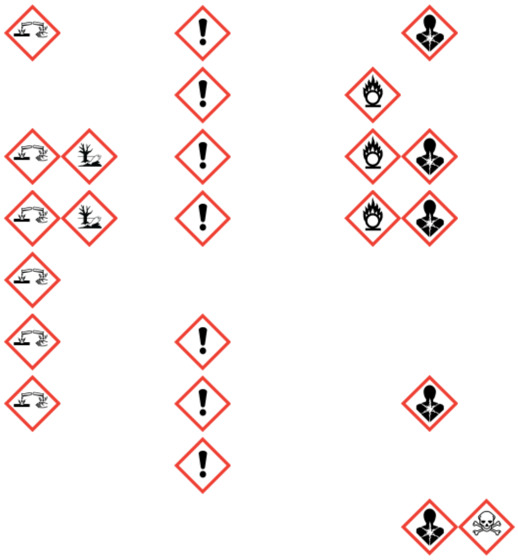

Note that this experiment involves a controlled combustion. Appropriate care and precautions should be taken.

Introduction

Magnetic nanomaterials have a vast array of potential and realised applications, ranging from data storage to biomedical imaging. Among the most versatile and widely studied classes of magnetic nanomaterials are the spinel-type oxides, with the general formula AB_2O_4. The structure may be considered as a cubic close-packed array of oxide ions where A^{2+} occupies 1/8 of the tetrahedral sites and B^{3+} occupies 1/2 of the octahedral sites. This is the *normal* spinel structure. *Inverse* spinels may be described by the general formula $B(AB)O_4$, whereby the A ions have “swapped” position with half of the B ions (indeed, spinels may also adopt structures between those of *normal* and *inverse* phases). The compositional and structural flexibility of the spinel oxides allows for an enormous degree of tuneability in their magnetic properties. This, in combination with nanoscale effects such as high surface areas, superparamagnetism, and dispersibility, gives such magnetic materials their wide range of applications. In this experiment you will prepare a number of transition metal ferrite spinels (MFe_2O_4; M= Zn, Ni, Co) by the sol-gel combustion method, and an aqueous ferrofluid by a co-precipitation reaction.

Sol-gel combustion is a (relatively) low temperature, energy efficient, self-propagating method of materials synthesis that involves a spontaneous exothermic redox reaction between a metal nitrate and an organic fuel. The reaction combines sol-gel chemistry, which typically yields poorly crystalline product, with combustion ; using the heat produced from the reaction of the oxidant (nitrate) and reductant (organic fuel) coming into contact in air to drive crystallisation. During the sol-gel process metal ions in solution are intimately mixed by formation of a gel network often mediated by chelating organic ligands, producing precursors of well-defined stoichiometry suitable for conversion to the desired (mixed) metal oxide, typically by post-synthetic drying and calcination steps. In sol-gel combustion syntheses as the temperature increases, any water present evaporates and spontaneous

combustion propagates throughout the reaction mixture, yielding fine particle metal oxides with the liberation of gaseous side-products.

The reaction can be broken down into three major steps:

1. Hydrolysis: metal complexes → metal hydroxides
2. Condensation: sol → gel
3. Drying/combustion: formation of final product

The resultant powders are typically porous due to the liberation of gaseous products upon reaction.

The most efficient auto-combustion reaction is achieved when the oxidiser:fuel ratio, known as the **equivalence ratio (φ_C),** is unity. This ratio, (φ_C), is calculated by balancing the elemental oxidising and reducing valencies of the compounds utilised in combustion, as follows:

$$\varphi_C = \frac{total\ oxidising\ valency\ of\ reactants}{-\ total\ reducing\ valency\ of\ reactants}$$

Where the valency of each atom is taken as;[4] N = 0; O = -2*; H = +1; C = +4; M^{n+} = n^+ (+2, +3, +4, etc). **Note:** O^{2-} is taken to be the only oxidising element.

Ferrofluids are stable, colloidal dispersions of magnetic nanoparticles, in which surface modification of very small nanoparticles with surfactants provides sufficient interparticle repulsion to prevent agglomeration of the nanoparticles, resulting in a magnetic fluid. Mild sol-gel co-precipitation reactions can produce sufficiently small spinel nanoparticles, which may be readily modified with hydrophobic or hydrophilic surfactants, to yield magnetic fluids in different solvents.

Part 1.1 – Synthesis and characterisation of transition metal ferrite spinels

Prepare stock solutions of zinc(II) nitrate hexahydrate (1.840 g), nickel nitrate hexahydrate (1.801 g), cobalt nitrate hexahydrate (1.804 g) iron(III) nitrate nonahydrate (10.00 g) and citric acid (5.278 g) each in 50 mL volumetric flasks using distilled or deionised water.

Into a large beaker (500 mL), pipette 20 mL of zinc nitrate, and 10 mL each of both iron nitrate and citric acid solutions. Allow this solution to stir before heating to ensure thorough mixing. Remove the stir bar and heat the mixture to 300 °C. Allow the mixture sufficient time for reaction to reach completion (approx. 25 mins). Observe what happens upon total evaporation of water and formation of product. **Ensure this is done at the back of a fume hood as nitrous oxides and other gases will evolve from the reacting mixture (a sufficiently large clock glass should be kept nearby to cover the mouth of the beaker if the reaction becomes particularly violent).** Allow the beaker to cool before collecting and grinding your product to a fine powder using a pestle and mortar.

Repeat the above procedure using the nickel and cobalt nitrate solutions in place of zinc, mixing 20 mL of each with iron nitrate and citric acid as before. Keep your three products for later magnetic measurements on the magnetic susceptibility balance outlined in Part 1.2.

Record the yield of each product.

Note the response of each product to the strong magnets provided.

Representative powder X-ray diffraction patterns for each product have been provided. From these calculate the unit cell parameters and crystallite sizes of each product (**Note – the strongest peak in each pattern is best suited for these calculations**).

Transmission electron micrographs have also been provided for each sample.

Part 1.2 – Magnetic measurements

The strongly magnetic nature of these samples necessitates a modification to the normal procedures for magnetic measurements outlined at the end of this manual. Here the samples must be diluted in a diamagnetic diluent, KBr. To do so grind approximately 0.01 g of each product with 0.99 g of KBr to a fine, consistent powder (take note of the precise masses used). From here the procedures outlined in the "Magnetic Measurements" section of the manual may be followed, but the range knob on the balance must be on the × 10 scale. A sample of unadulterated KBr should be measured as well.

To account for the dilution in subsequent calculations one may use the following relationship to determine χ_g:

$$\chi_s = \frac{m_1}{m_1 + m_0}\chi_g + \frac{m_0}{m_1 + m_0}\chi_0$$

where χ_s is the mass susceptibility of the diluted sample, χ_g and χ_0 are the mass susceptibilities of the product and diluent, respectively, m_1 is the mass of product, and m_0 is the mass of diluent.

Report your readings, χ_g, and μ_{eff} for each sample.

Part 2 – Preparation of an aqueous ferrofluid

To prepare a ferrofluid sample the magnetic core must first be produced, in this case, magnetite, Fe_3O_4. Mix 5 cm^3 of 2 M $FeCl_2 \cdot 4H_2O$ in 2 M HCl and 20 mL of 1 M $FeCl_3 \cdot 6H_2O$ in 2 M HCl. With constant stirring using a glass rod, slowly (dropwise) add ~ 10 – 13 mL of concentrated ammonia (**work in the fumehood**). This should result in a black suspension. Stop stirring and allow the magnetite to settle. Decant off as much liquid as possible. Wash the product several times by addition of water followed by settling and decanting (this may be sped up by use of a magnet or by leaving the beaker on the stirplate). Once as much liquid has been removed as possible add small aliquots of tetramethylammonium hydroxide to the magnetite while mixing with a glass rod. The product should achieve a fluidic state and respond to a magnet by "spiking". A portion of the fluid may be transferred to a plastic weigh boat to make this manipulation easier.

Note that achieving a high quality ferrofluid by this process can be quite difficult.

Include a photograph of your ferrofluid in the presence of a magnetic field.

Data checklist

**You will need to collect the following data:**	**The following data has been provided for you:**
Yields of spinels<br>Magnetic measurements of spinels<br>Photograph(s) of your ferrofluid	Powder X-ray diffraction patterns of zinc, nickel, and cobalt ferrites<br>Transmission electron micrographs of zinc, nickel, and cobalt ferrites

Assessment

For this experiment, you will be assessed on the following:

- Overall standard of lab report,
- Yields of sol-gel combustion products,
- Analysis and interpretation of XRD patterns (unit cell parameters and crystallite size) and TEM images and related discussion,
- Magnetic measurements,
- Ferrofluid quality.

Your lab report should also address the following specific points:

1. Explain whether you would expect the products of part 1 to be normal or inverse spinels, giving your justification.
2. Comment on your observations of the response of each product to the magnets, and on your magnetic measurement results. Is this approach to magnetic measurement suitable for these materials?
3. Provide a balanced equation for the sol-gel combustion synthesis of $Zn_{0.5}Ni_{0.5}Fe_2O_4$ from the corresponding metal nitrate salts using oxalyl dihydrazide ($C_2H_6N_4O_2$) as the fuel.

Magnetic Moment Measurements

Introduction

Transition metals, by definition, have at least one oxidation state with an incomplete d or f subshell. Since electrons spin and generate a magnetic field, the magnetic properties of transition metals are of great interest in determining the metal's oxidation state, electronic configuration and magnetic properties. Most organic and main group compounds have all their electrons paired. Such molecules are diamagnetic and have very small magnetic moments. Many transition metal compounds, however, have one or more unpaired electrons, and are termed paramagnetic.

Measurement of the magnetic susceptibility, χ, of a compound can yield information of the number of unpaired electrons, and the stereochemistry. When measured over a temperature range, detailed information of the electronic structure can be obtained.

Calculating magnetic susceptibility: Evans method

This method works in the opposite fashion to the traditional Gouy method. Instead of weighing a mass change in the sample and sample tube, it weighs an equal and opposite force on the magnet while the sample is in a fixed position.

Step 1: Calibration of Gouy tube

The calibration constant, C, can be determined using the following relationship:

$$C = \frac{\chi_g m \times 10^9}{l(R - R_0)} = 1.05$$

It has already been measured in the lab using a series of calibrants, which are complexes such as $HgCo(SCN)_4$ (χ_g = 16.44 x 10^{-6} at 293.2 K) and $[Ni(en)_3][S_2O_3]$ (χ_g = 11.04 x 10^{-6} at 293.2 K) for which the χ_g is known.

Step 2: Calculation of the gram susceptibility, χ_g

Knowing the calibration constant allows the gram susceptibility to be calculated according to the following equation:

$$\chi_g = \frac{Cl(R - R_0)}{m \times 10^9}$$

where C is a calibration constant, R is the reading obtained for tube plus sample, R_0 is the empty tube reading (normally negative), I is the sample length (in cm), m is the sample mass (in grams).

Step 3: Calculation of the diamagnetic susceptibility, χ_{dia}

A diamagnetic correction must be made to take into account magnetic contributions by the ligands attached to the metal centre. The magnetic contribution of an atom or ligand is expressed in terms of its Pascal's constant.[1] These constants, for a series of atoms, functional groups and ligands, are given in Table 1 below. In some instances one type of atom has two different values, depending on it's chemical nature:

e.g. for an ether or alcohol, χ_{dia} (O) = - 4.61 x 10^{-6}

e.g. for a ketone or aldehyde, χ_{dia} (O) = + 1.73 x 10^{-6}

Pascal's constant for each atom is multiplied by the number of each type of atom in the ligand and all the contributions are added together. Constitutive corrections are then made for the contribution by electrons in aromatic systems and double bonds (see Table 2, below).

e.g. $[Ni(PPh_3)_2Cl_2]$

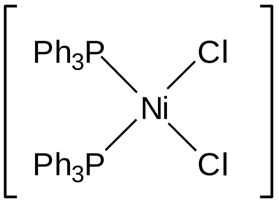

$C_{36}H_{30}Cl_2NiP_2$

MW = 654.18

First calculate χ_{dia} for all atoms in the complex:
(if one type of atom is missing, do the calculation without it – it will make a negligible difference)

36C + 30H + 2Cl + Ni + 2P

(36 x –6x10^{-6}) + (30 x –2.93x10^{-6}) + (2 x +-23.4x10^{-6}) + (1 x –13x10^{-6}) + (2 x +-26.3x10^{-6})
= -4.163 x 10^{-4}

Constitutive correction: 36 aromatic carbons in 2 PPh_3 ligands

= (36 x +0.24 x 10^{-6}) = + 8.64 x 10^{-6}

So χ_{dia} of $[Ni(PPh_3)_2Cl_2]$ is –4.0766 x 10^{-4}

Step 4: calculation of the magnetic susceptibility, χ_m

$$\chi_m = \chi_g \times MW - \chi_{dia}$$ (MW = molecular weight)

Step 5: calculation of magnetic moment, μ_{eff}

$$\mu_{eff} = 2.83\sqrt{\chi_m T}\ \text{BM}$$

(use the temperature, in Kelvins, in the lab when you measure)

[1] G. A. Bain and J. F. Berry, *J. Chem. Edu.*, 2008, **85**, 532

Table S1: Diamagnetic Corrections (all values × 10^{-6} /g atom)

**Pascal's constants**			
Na^{+}	-6.8	H	-2.93
K^{+}	-14.9	C	-6.00
Cs^{+}	-35.0	P	-26.3
NH_4^{+}	-13.0	S	-15
F^{-}	-9.1	N ring	-4.61
Cl^{-}	-23.4	N open chain	-5.57
Br^{-}	-34.6	N mono-amide	-1.54
I^{-}	-50.6	N diamide imide	-2.11
Co, Fe, Ni	-13	O ether alcohol	-4.61
NO_3^{-}	-18.9	O ketone aldehyde	+1.73
ClO_4^{-}	-32.0		

Table S2: Constitutive corrections (all values x 10^{-6} /g atom)

**Constitutive corrections**			
C=C	+5.5	bipyridine	-105
C=C-C=C	+10.6	1,10-phenanthroline	-128
C≡C	+0.8	$salen^{2-}$	-182
M=N	+1.8	diethyldithiocarbamate	-95
C=N-R	+8.2	H_2O	-13
C in benzene	+0.24	pyridine	-49
C-Cl	+3.1	dimethylsulfoxide	-43
C-Br	+4.1	oxalate	-25
C-I	+4.1		
VO^{2+}	-12.5		

1. Turn the RANGE knob to the ×1 scale and allow 10 mins for the apparatus to warm up. If the balance is to be used frequently, it is recommended that it be left on continuously.
2. Determine the mass of the empty sample tube on the analytical balance
3. Adjust the zero knob until the display reads 000
4. Place the empty sample tube into the tube guide and take the reading R_0
5. Pack the sample tube with sample. Solid samples are first ground to a very fine powder in an agate pestle and mortar and packed by repeated tapping on a wooden surface (fill in 1 cm portions). The sample length, l, should be in the range 2.5-3.5 cm. Ensure that the surface of the sample is horizontal
6. Determine the sample mass, m, in grams by weighing on the analytical balance
7. Measure the length, l, in centimetres
8. Place the packed sample tube into the tube guide and take the reading, R. If the display goes off the scale, turn the range knob to the x 10 scale, re-zero and multiply the reading by 10
9. In order to eliminate packing errors, tip out the powder, repack, reweigh (on the analytical balance), measure the length and re-measure R.
