# Supplementary material for: Introducing Materials Science: Experimenting with Magnetic Nanomaterials in the Undergraduate Chemistry Laboratory
Source: J Chem Educ. 2023 May 8;100(6):2387–93. doi: 10.1021/acs.jchemed.3c00121 (PMC10269328; doi:10.1021/acs.jchemed.3c00121)
Supplement: Supplementary file 2 — ed3c00121_si_002.pdf [file ed3c00121_si_002.pdf]

Supporting Information

Introducing Materials Science: Experimenting with Magnetic Nanomaterials in the Undergraduate Chemistry Laboratory

Annie Regan,[1,2] John O'Donoghue,[1] Carl Poree,[1] and Peter W. Dunne.[1] *

1. School of Chemistry, Trinity College Dublin, College Green, Dublin 2, Ireland

2. CDT ACM, AMBER, Trinity College Dublin, College Green, Dublin 2, Ireland

*Corresponding author, email: p.w.dunne@tcd.ie

Notes for Instructors

Yields:

Either metal salt may be considered the limiting reagent. Yields should be close to 100% (plus or minus).

XRD/TEM:

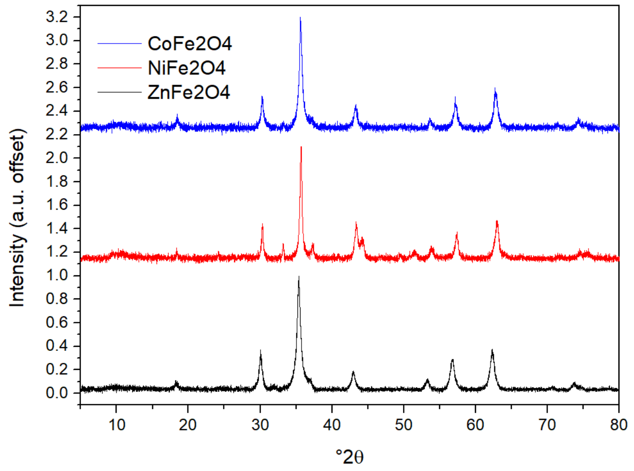

Figure S1. XRD of spinel ferrites.

Most intense peak is the (311). This will be needed for unit cell parameter determination. The weaker earlier peaks and impurities in the nickel ferrite may catch students out here, but they should know to compare to CIFs/predicted patterns.

TEMs should be presented and discussed; porosity and particle size should be mentioned, but no great detail needed here.

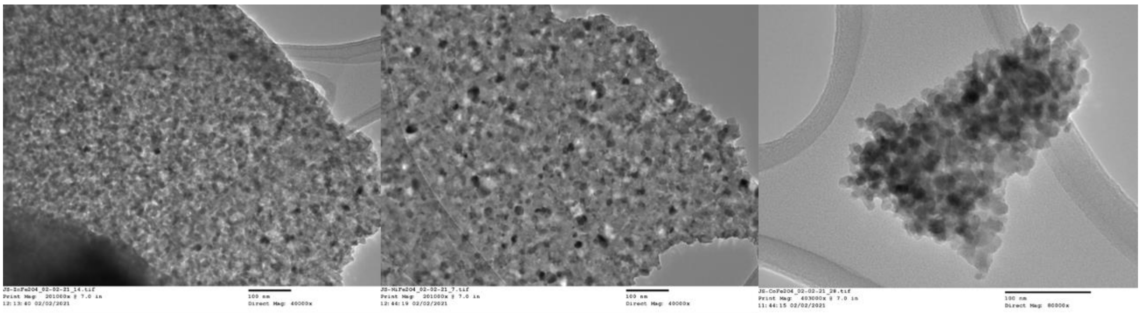

Figure S2. TEM of spinel ferrites.

Table S1. Sample values

Sample	Unit cell parameter (Å)	Crystallite size (nm)	R (x10)	X_g	μ_{eff}
$ZnFe_2O_4$	8.41	20	91	1.36E-05	2.78
$NiFe_2O_4$	8.34	55	493	6.98E-05	6.28
$CoFe_2O_4$	8.35	30	780	1.01E-04	7.52
KBr			-11	-6.3E-09	6.28

Zinc ferrite least magnetic. This approach is not very suitable – lots of sources of error, and doesn't give much useful information but is good for comparative purposes.

Ferrofluid should give clear spikes (this is very tricky to get right so a low weighting here 5-10% of report overall)

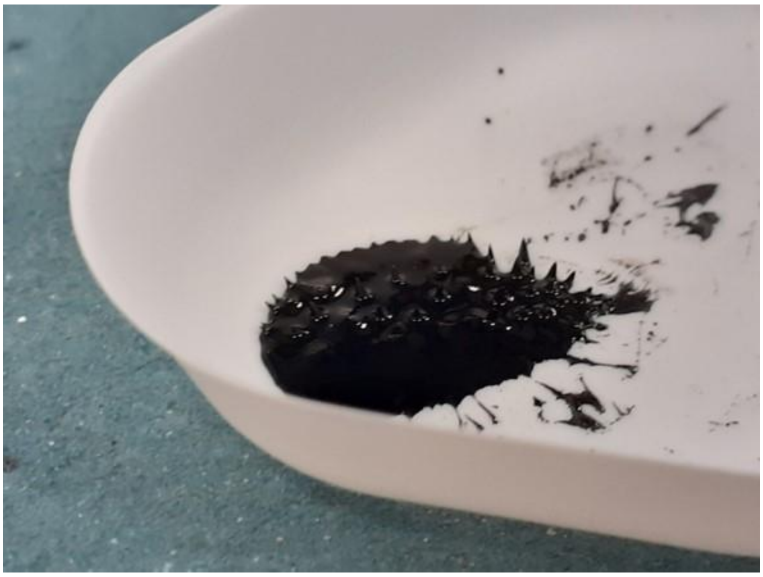

Figure S3. Example of a ferrofluid spiking in response to an external magnet.

ODH question: 0.5 Zn, 0.5 Ni, 2 Fe, 4 ODH
