# Supplementary material for: Introducing Materials Science: Experimenting with Magnetic Nanomaterials in the Undergraduate Chemistry Laboratory
Source: J Chem Educ. 2023 May 8;100(6):2387–93. doi: 10.1021/acs.jchemed.3c00121 (PMC10269328; doi:10.1021/acs.jchemed.3c00121)
Supplement: Supplementary file 3 — ed3c00121_si_003.pdf [file ed3c00121_si_003.pdf]

Supporting Information

Introducing Materials Science: Experimenting with Magnetic Nanomaterials in the Undergraduate Laboratory

Annie Regan,[1,2] John O'Donoghue,[1] Carl Poree,[1] and Peter W. Dunne[1] *

1. School of Chemistry, Trinity College Dublin, College Green, Dublin 2, Ireland

2. CDT ACM, AMBER, Trinity College Dublin, College Green, Dublin 2, Ireland

*Corresponding author, email: p.w.dunne@tcd.ie

Anonymous Student Survey

Experiment H: Magnetic Nanoparticles - Anonymous Student Survey

Thank you for being involved in this study. Experiment H was one of two new experiments added to the JS inorganic practical laboratory course in 2020/21. The experiment was designed to introduce students to new topics and characterization methods that they may not otherwise encounter in a lab setting during their undergraduate studies.

This survey should only take you about 5 mins.

What will the study involve? The aim of this anonymous survey is to assess student satisfaction with the experiment and the effect it may have had on their understanding of any topics covered. The survey will consist of various questions that will allow us to assess the merits of this experiment for learning and provide us with valuable information to improve it based on your feedback.

Why have you been asked to take part? You have been asked because you completed Experiment H during your JS inorganic practical course at the School of Chemistry in Trinity College Dublin (TCD).

Do you have to take part? No, participation is entirely voluntary and you can withdraw from this study now or after you begin. After you submit your responses to this survey, you can withdraw your data any time in the next 2 weeks by emailing the researcher at anregan@tcd.ie with the date and time that you completed the survey. If you choose to withdraw, all of your submitted data will be deleted.

Will your participation in the study be kept confidential? Yes. We will ensure that no clues to your identity appear in the write-up. Any extracts from what you say that are quoted in the write-up will be entirely anonymous.

What will happen to the information that you give? Each survey response will only be identified by an anonymous participant number related to the Date & Time of completion, contact information will not be asked for or stored. The anonymous data file will only be seen by members of the research team. The data will be stored on an encrypted computer for a maximum period of 5 years and then destroyed.

What will happen to the results? The results will be used to improve the offering in the future and may be published in a research article in an academic journal or presented at conferences as a poster or presentation. No clues to your identity will appear in any publicized results.

What are the possible disadvantages of taking part? We don't envisage any negative consequences for you in taking part. We advise that you take time to ready this information and consider everything before consenting to taking part. If at any stage you feel distressed by any of the questions, please discontinue the survey. Any responses that you have given will not be stored.

If you need any further information, you can contact Annie Regan (anregan@tcd.ie) or Prof. Peter Dunne (p.w.dunne@tcd.ie)

* Required

1. If you agree to take part in the study, please click 'I Consent' below and then begin the survey.

   By doing so, you acknowledge that 'I understand the purpose of the study and am participating voluntarily. I am aware I can withdraw at any time, even after starting the survey. I understand that my anonymity will be ensured. I understand that I can withdraw permission to use my data within two weeks of completing each survey by emailing the researcher, in which case my response will be deleted.'

   By clicking consent you also confirm that you completed Experiment H during the JS inorganic lab practical sessions. *

   ○ I Consent

   ○ I Do Not Consent

Survey Questions

Reminder: Participation in this survey is entirely voluntary and you can withdraw from this study at any stage and have your data removed up to 2 weeks after submission (please note the exact date and time when you submit the survey).

2. How familiar were you with the field of '**materials science**' prior to completing this experiment?

1	2	3	4	5

Not Familiar at All — Very Familar

3. Please rate the experiment overall in terms of how **INTERESTING** you found it. *

1	2	3	4	5

Not interesting at all. — Extremely interesting.

4. Please rate the experiment overall in terms of how **USEFUL** you found it. *

1	2	3	4	5

Not useful at all. — Extremely useful.

5. Can you provide some details of the **materials science concepts** that you used during this experiment? *

6. Can you provide some details of the **synthetic techniques** that you used during this experiment? *

7. Can you provide some details of the types of **analysis** that you used during this experiment? *

8. The time given to complete **Part 1 (Sol-Gel)** of this experiment was: *

1	2	3	4	5

Not Sufficient Sufficient

9. The time given to complete **Part 2 (Ferrofluid)** of this experiment was: *

1	2	3	4	5

Not Sufficient Sufficient

10. The time given to complete this **ENTIRE** experiment was: *

1	2	3	4	5

Not Sufficient Sufficient

11. Please select the option that best describes **how challenging** you found each of the following: *

	Difficult	Relatively Difficult	Similar to other experiment s	Relatively Easy	Easy
Carrying out the lab practical overall.	○	○	○	○	○
Carrying out part 1 (sol-gel) of the practical.	○	○	○	○	○
Carrying out part 2 (ferrofluid) of the practical.	○	○	○	○	○
This is a concentration test, please select "Relatively Difficult" here.	○	○	○	○	○
Writing the lab report	○	○	○	○	○

12. Is there anything else you felt you learned during this experiment that you considered useful? (Optional)

13. If anything, what would you change about this experiment? (Optional)

This content is neither created nor endorsed by Microsoft. The data you submit will be sent to the form owner.

Microsoft Forms
