# Supplementary figures and images for: Introducing Materials Science: Experimenting with Magnetic Nanomaterials in the Undergraduate Chemistry Laboratory
Source: J Chem Educ. 2023 May 8;100(6):2387–93. doi: 10.1021/acs.jchemed.3c00121 (PMC10269328; doi:10.1021/acs.jchemed.3c00121)

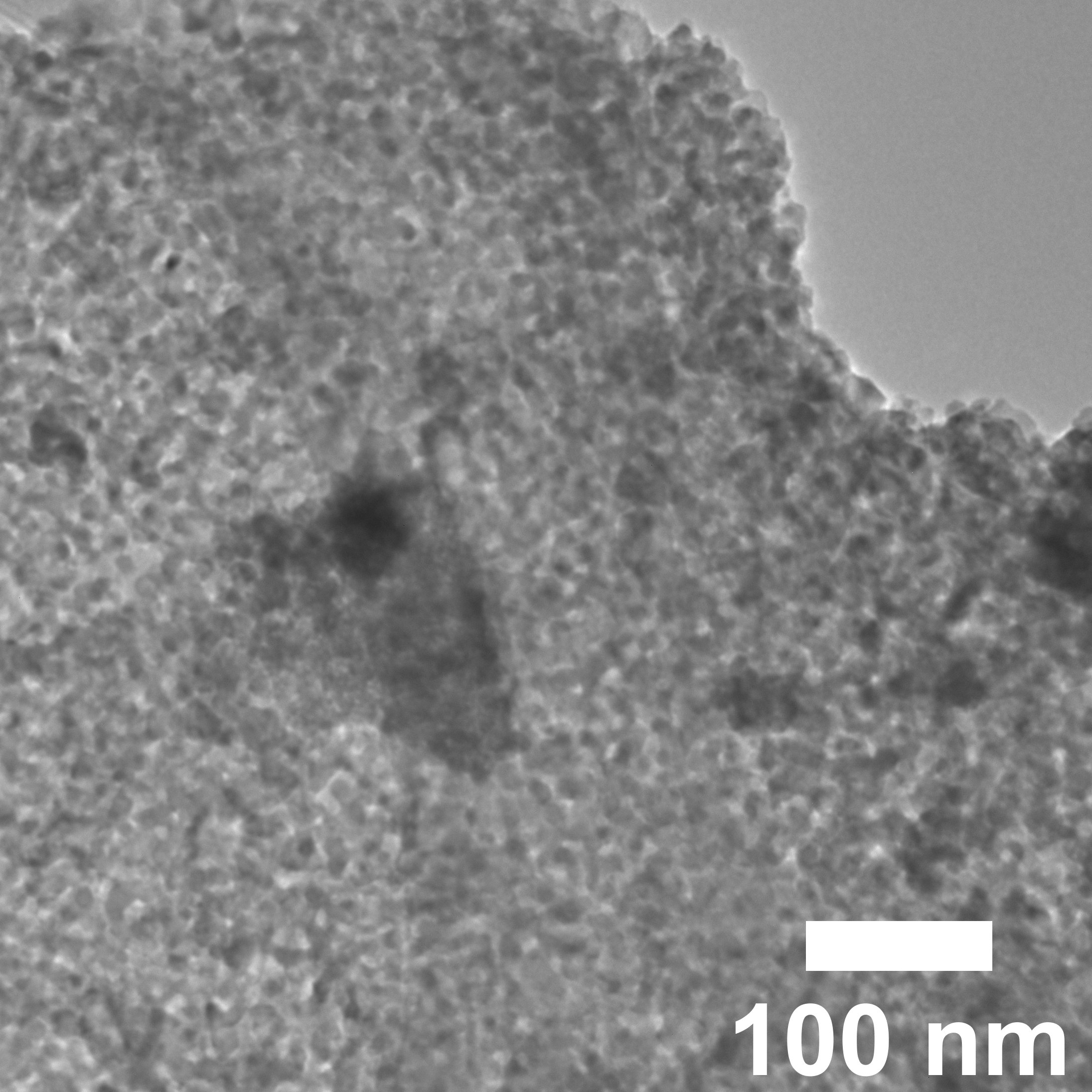

Supplement: Supplementary file 7 — ed3c00121_si_007.jpg [file ed3c00121_si_007.jpg]

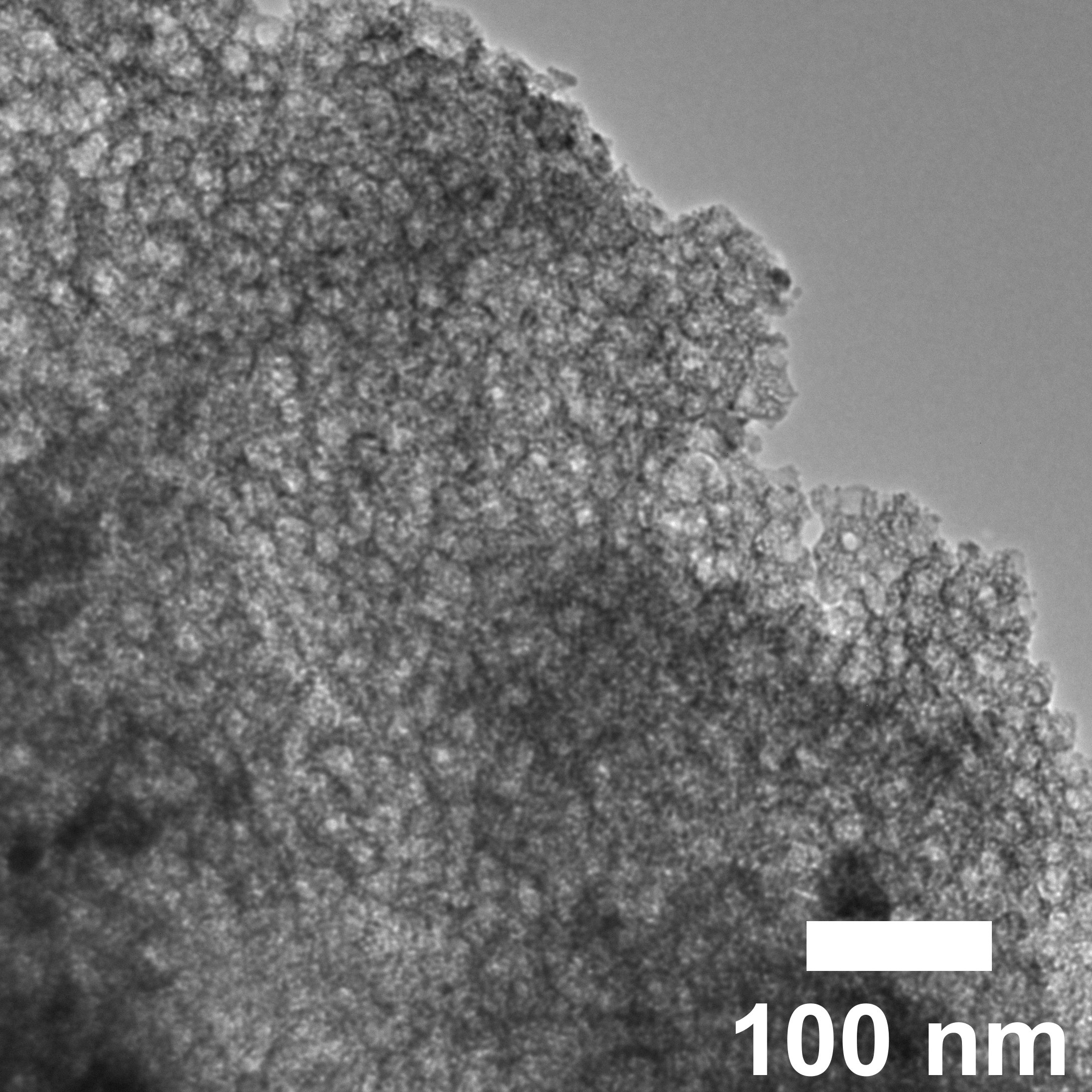

Supplement: Supplementary file 8 — ed3c00121_si_008.jpg [file ed3c00121_si_008.jpg]

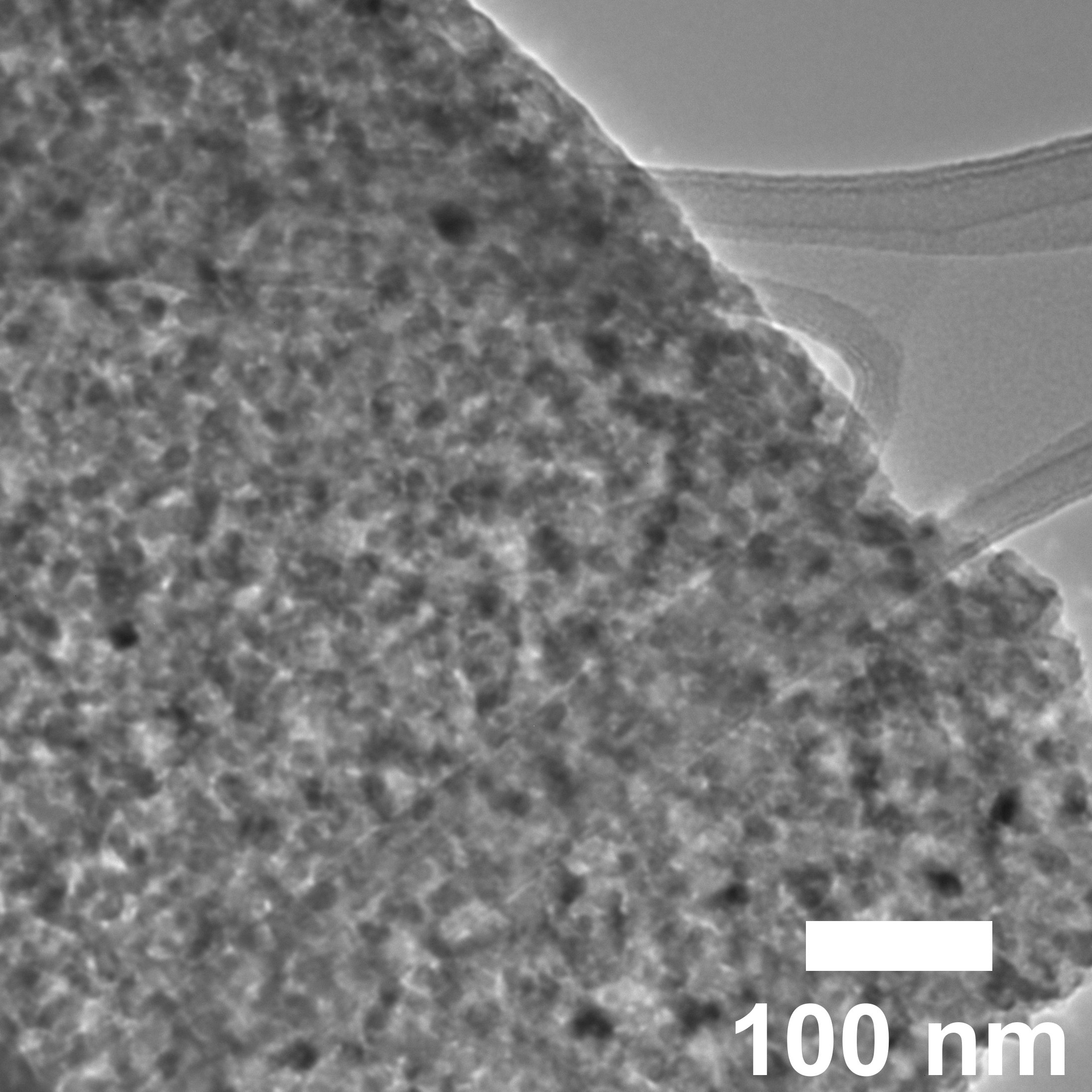

Supplement: Supplementary file 9 — ed3c00121_si_009.tif [file ed3c00121_si_009.tif]
